# Supplementary material for: Factors associated with infant mortality in Nigeria: A scoping review
Source: PLoS One. 2023 Nov 15;18(11):e0294434. doi: 10.1371/journal.pone.0294434 (PMC10650982; doi:10.1371/journal.pone.0294434)
Supplement: S1 Table — (DOCX) [file pone.0294434.s001.docx]

**Supplementary Information**

**S1 Table 1: Search strategy applied to databases.**

| **Concepts** | Infant; mortality; Nigeria |
| --- | --- |
| **Synonyms/other keywords** | Infant; “newborn”; baby; neonate; neonatal; postneonatal.  mortality; fatality; death; dying; loss; Nigeria; Nigerian; and  Nigeria; “Federal Republic of Nigeria” |
| **Truncation** | morta*; fatal* |
| **MeSH words** | - Infant mortality - Neonatal mortality - Postneonatal mortality |
| **Year** | All publications up to 2022 |
| **Databases** | 1. PubMed 2. Ovid MEDLINE 3. CINAHL Complete 4. Web of Science 5. Sabinet African Journal |
| **Studies to be included** | No limitation for type of study |
| **Inclusion criteria** | Articles addressing the phenomenon of infant mortality in any geographical region in Nigeria will be included in this study.  The population of interest will be infants or babies, less than one year of age (i.e., birth to 365 days), this includes neonates, perinates and postneonates.  The context of all included studies will be Nigeria.  Only publications written in English language will be eligible. |
| **Exclusion criteria** | Studies with participants that are more than one year of age will be excluded. Also, any participant/study from a country other than Nigeria will be excluded.  Publications will be excluded if they are not available as free full text. |

**S1 Table 2: Search History on PubMed**

| Search | Query | Results |
| --- | --- | --- |
| #5 | Search: ((#1) AND (#2)) AND (#3) Filters: English | 3,206 |
| #4 | Search: ((#1) AND (#2)) AND (#3) | 3,231 |
| #3 | Search: Nigeria OR Nigerian OR "Federal Republic of Nigeria" | 66,094 |
| #2 | Search: Mortality OR Mortalities OR Fatality OR Death OR Dying OR Loss | 3,302,063 |
| #1 | Search: Infant OR Newborn OR "new born" OR Baby OR Neonate OR Neonatal OR Postneonatal OR Perinatal OR Fetal OR Foetal OR Postnatal | 1,973,747 |

**S1 Table 3: Search History on MEDLINE (Ovid)**

| [#](https://ovidsp-dc1-ovid-com.proxy.lib.ul.ie/ovid-b/ovidweb.cgi?&S=FDICFPACNOACAGGHKPNJIFPKFCDCAA00&Sort+Sets=descending) | Searches | Results |
| --- | --- | --- |
| 1 | (Infant or Newborn or "new born" or Baby or Neonate or Neonatal or Postneonatal or Perinatal or Fetal or Foetal or Postnatal).mp. [mp=title, abstract, original title, name of substance word, subject heading word, floating sub-heading word, keyword heading word, organism supplementary concept word, protocol supplementary concept word, rare disease supplementary concept word, unique identifier, synonyms] | 1837429 |
| 2 | (Mortality or Mortalities or Fatality or Death or Dying or Loss).mp. [mp=title, abstract, original title, name of substance word, subject heading word, floating sub-heading word, keyword heading word, organism supplementary concept word, protocol supplementary concept word, rare disease supplementary concept word, unique identifier, synonyms] | 2991006 |
| 3 | (Nigeria or Nigerian or "Federal Republic of Nigeria").mp. [mp=title, abstract, original title, name of substance word, subject heading word, floating sub-heading word, keyword heading word, organism supplementary concept word, protocol supplementary concept word, rare disease supplementary concept word, unique identifier, synonyms] | 44669 |
| 4 | 1 and 2 and 3 | 2491 |
| 5 | limit 4 to English language | 2466 |

**S1 Table 4: Search History for CINAHL Complete**

| ID | Search Terms | Search Options | Results |
| --- | --- | --- | --- |
| S5 | S1 AND S2 AND S3 | **Expanders** - Apply equivalent subjects  **Narrow by Language**: - English  **Search modes -** Boolean/Phrase | (592) |
| S4 | S1 AND S2 AND S3 | **Expanders** - Apply equivalent subjects  **Search modes** - Boolean/Phrase | (592) |
| S3 | Nigeria OR Nigerian OR Federal Republic of Nigeria | **Expanders** - Apply equivalent subjects  **Search modes** - Boolean/Phrase | (11,112) |
| S2 | Mortality OR Mortalities OR Fatality OR Death OR Dying OR Loss | **Expanders** - Apply equivalent subjects  **Search modes** - Boolean/Phrase | (692,741) |
| S1 | Infant OR Newborn OR "new born" OR Baby OR Neonate OR Neonatal OR Postneonatal OR Perinatal OR Fetal OR Foetal OR Postnatal | **Expanders** - Apply equivalent subjects  **Search modes** - Boolean/Phrase | (450,362) |

**S1 Table 5: Web of Science Search**

| # | Query | Results |
| --- | --- | --- |
| 5 | #1 AND #2 AND #3 and English (Languages) | 1,753 |
| 4 | #1 AND #2 AND #3 | 1,753 |
| 3 | ALL= (Nigeria OR Nigerian OR "Federal Republic of Nigeria") | 151,032 |
| 2 | ALL= (Mortality OR Mortalities OR Fatality OR Death OR Dying OR Loss) | 4,595,985 |
| 1 | **ALL=(Infant OR Newborn OR “new born” OR Baby OR Neonate OR Neonatal OR Postneonatal OR Perinatal OR Fetal OR Foetal OR postnatal)** | 1,209,757 |

**S1 Table 6:** **Search strategy on Sabinet African Journal (**[**https://journals.co.za/**](https://journals.co.za/)**)**

| Search Fields | Search terms | Result |
| --- | --- | --- |
| Field 1 | **Publication Title:** Infant OR Newborn OR "new born" OR Baby OR Neonate OR Neonatal OR Postneonatal OR Perinatal OR Fetal OR Foetal OR Postnatal | 108 |
| Field 2 | Mortality OR Mortalities OR Fatality OR Death OR Dying OR Loss | 108 |
| Field 3 | Nigeria OR Nigerian OR "Federal Republic of Nigeria | 108 |
