# Supplementary material for: Factors associated with infant mortality in Nigeria: A scoping review
Source: PLoS One. 2023 Nov 15;18(11):e0294434. doi: 10.1371/journal.pone.0294434 (PMC10650982; doi:10.1371/journal.pone.0294434)
Supplement: S1 Appendix — (DOCX) [file pone.0294434.s002.docx]

# Factors affecting infant mortality in Nigeria: A scoping review protocol.

# Introduction:

An improvement in life expectancy for any country reflects a significant reduction in infant mortality (1). The number of infants that die before their first-year birthday for every 1,000 live births within a given year is known as Infant Mortality Rate (IMR). Infant mortality remains a public health issue even though the past two decades have shown significant progress in the reduction of infant deaths worldwide i.e., from 65 deaths per 1,000 live births in 1990 to 27.4 infant deaths per 1,000 live births in 2020 (2). However, low and middle-income countries (LIMCs) still record very high rates of infant deaths, most especially the sub-Saharan African (SSA) region with an average infant mortality rate (IMR) of 50.26 deaths in 2020 (2). Hug et al. (2019) state that the risk of an infant dying in an African Region is six times higher than in the European Region (3). Out of the 1.96 million infants that died in SSA, Nigeria recorded an estimated 548,116 infant deaths in 2019, representing 28% of total infants that died in SSA in 2019 (4).

As a significant indicator for a country’s socio-economic development index, IMR is used to assess the level of improvement in child health. A global target under the Sustainable Development Goals (SDGs)— Goal 3.2 is to end avoidable deaths of children under 5 years of age to at least 25 per 1,000 live births by 2030 (5). However, eight years to the deadline, Nigeria’s hope of achieving this goal seems doubtful as the rate of decline has been slow, from 124.6 deaths in 1990 to 72.2 deaths per 1,000 live births in 2020 (2). When compared to other countries in SSA e.g., South Africa, Kenya and Ghana, this progress seems inadequate as these countries are already close to achieving SDG 3.2, with respective IMR of 28, 31 and 35 deaths per 1,000 live births (6).

Despite being one of the largest economies in Africa with an estimated GDP of $448 billion in 2019, and a population of over 200 million people, Nigeria keeps dragging back Africa’s socioeconomic indicators, especially child mortality (7). Several studies have traced the high mortality rates of infants in Nigeria to demographic and socio-economic factors with the most common factors being poverty/socioeconomic status and poor health service delivery (6, 8). The slow decline in infant mortality rates in Nigeria raises questions about the barriers that affect the reduction of infant mortality despite the number of interventions and policies that have been employed to tackle the challenge.

Different studies have been conducted on the various factors affecting infant mortality with an emphasis on the characteristics of both mother and child. A conceptual framework developed by Matteson et al. (1998), categorized infant mortality risk factors into personal risk factors as a mother’s social, demographic and medical risk factors and contextual factors at the community level e.g., healthcare infrastructure, welfare expenditures, poverty rate and urbanization (9). Using the three-delays model to estimate the burden of social factors on neonatal deaths, Upadhyay *et al.* (2014) concluded that a delay in seeking care (level 1 delay) and a delay in receiving quality treatment at a health facility (level 3 delay) significantly contributed to neonatal mortality as against a delay in reaching a health facility (level 2 delay) (10). A review by Louangpradith *et al.* (2020) suggests three categories of predictive factors for infant mortality: distal factors (i.e. socioeconomic and demographic factors), intermediate factors such as maternal, reproductive and healthcare delivery factors and lastly, proximal (neonatal) factors (11). Furthermore, by adopting the conceptual model of health capability (CMHC) framework for the analysis of different social determinants of infant mortality, Bugelli *et al.* (2021) revealed that infant mortality rate is affected by social and environmental conversion factors such as employment rate, educational status, quality of prenatal care and access to health professionals (12). The conclusions from these studies suggest that infant mortality rates and birth outcomes are linked to maternal health and wellbeing (13).

An initial search of MEDLINE and PubMed databases was conducted, and no scoping review has been done on the topic. Thus, for this study, a scoping review was chosen to identify and map out existing literature on the factors affecting infant mortality in Nigeria and to summarize any gap in research regarding the health determinants that influence infant death and survival. The outcome of this review will be useful to policymakers and other health system stakeholders in Nigeria, to help plan evidence-based interventions that promote child well-being and facilitate the achievement of the SDG’s goal 3.2 by 2030.

**Review Question:**

The review question for this study is: What are the factors affecting infant mortality in Nigeria?

**Objectives of the review:**

Based on the framework by Arksey and O’Malley (14), this scoping review aims to identify and summarize the breadth of evidence reported on the factors affecting infant mortality in Nigeria. The results from this scoping review will help to develop evidence-based child health interventions and to specify a detailed research topic for a systematic review in future.

# Methodology

This protocol is designed in compliance with the Arksey and O’Malley framework, involving the following stages: defining the research question; identifying relevant literature; study selection; data extraction; and lastly, collating, summarizing, and reporting the results; (14). Additionally, the summary and reporting of this review will be guided by the checklist for the Preferred Reporting Items for Systematic reviews and Meta-Analyses extension for Scoping Reviews (PRISMA-ScR) (15). No ethical approval is required for this review as results will be based on existing published and publicly available literature. The proposed timeline for conducting this review includes initiating and finalizing the search strategy by May 2022, while data screening, extraction, and synthesis will take place from June 2022 with the scoping review being finalized by August 2022.

**Inclusion and Exclusion Criteria**

The inclusion criteria will follow the Population, Concept, and Context (PCC) framework.

**Population:** The population of interest are infants less than one year of age (i.e., birth to 365 days). Also, studies that address maternal factors and conditions will be included.

**Concept:** studies addressing the concept and factors affecting infant mortality will be included in this study.

**Context:** The context of all included studies will be Nigeria.

In addition, no limit will be applied to the publication date and type of study, as the aim of the review is to provide a broad overview of the concept of infant mortality in Nigeria. Only publications written in English language will be eligible.

**Exclusion Criteria:** Studies will be excluded if they are not available as full text and if they do not assess the factors affecting infant mortality. Most importantly, studies having participants from a country other than Nigeria will be excluded. Studies having population/participants that are more than one year of age will be excluded.

**Stage 1—Defining the research question.**

To achieve the overall objective of this study, the review question formulated for this study is as follows: What is known about the factors affecting infant mortality in Nigeria?

**Stage 2 —Identifying relevant literature.**

The review question will be assessed based on studies that are specific to the population, concept and context of interest. An extensive search of the literature will be carried out on the following electronic databases such as PubMed, Ovid MEDLINE, CABI Global Health, CINAHL, Web of Science and African Journal Online. Google scholar will be used to also search for grey literatures and scientific reports. Executive summaries and reports will be obtained from websites and databases of organizations that support child and maternal health such as WHO, UNICEF, Save the Children, Nigeria Ministry of health and other local sources.

The search strategy will include infant mortality and Nigeria. Keywords will be obtained from medical subject heading (MeSH) and from other references to avoid missing out any search terms. Following an initial search on PubMed, the search strategy combined the following key terms and Medical Subject Headings (MeSH) terms: Infant; newborn; baby; neonate; neonatal; fetal; foetal; perinatal; postneonatal; mortality; fatality; death; dying; loss; Nigeria; Nigerian; and “Federal Republic of Nigeria”. The initial search strategy is outlined in Table 1 below.

**Table 1:** **Initial Search strategy on PubMed**

| Search | Query | Results |
| --- | --- | --- |
| #4 | Search: ((#1) AND (#2)) AND (#3) | [3,195](https://pubmed.ncbi.nlm.nih.gov/?term=%28%28%231%29+AND+%28%232%29%29+AND+%28%233%29&sort=&size=200) |
| #3 | Search: Nigeria OR Nigerian OR "Federal Republic of Nigeria" | [65,913](https://pubmed.ncbi.nlm.nih.gov/?term=Nigeria+OR+Nigerian+OR+%22Federal+Republic+of+Nigeria%22&sort=&size=200) |
| #2 | Search: mortality OR mortalities OR fatality OR death OR dying OR loss | [3,295,971](https://pubmed.ncbi.nlm.nih.gov/?term=mortality+OR+mortalities+OR+fatality+OR+death+OR+dying+OR+loss&sort=&size=200) |
| #1 | Search: Infant OR newborn OR baby OR neonate OR neonatal OR fetal OR foetal OR perinatal OR postneonatal | [1,922,658](https://pubmed.ncbi.nlm.nih.gov/?term=Infant+OR+newborn+OR+baby+OR+neonate+OR+neonatal+OR+fetal+OR+foetal+OR+perinatal+OR+postneonatal&sort=&size=200) |

**Stage 3 —Study selection**

Two independent reviewers will be involved in screening the articles using the inclusion criteria. The selection will involve two stages: first stage is the title and abstract screening and the second is a full-text review. After the search is complete, all citations and references to be included in the review will be uploaded into EndNote and Rayyan reference managers. The Endnote software will be used to organize and manage articles and references during the whole process. The software Rayyan will also be used to screen and select studies that meet the inclusion criteria. Duplicates and references will be excluded based on the title and abstracts and literature that do not answer the relevant review questions or meet the review objective. During the study selection process, the PRISMA flow diagram will be used and any reason for exclusion will be stated on the flow chart. This will be updated regularly until the review is complete.

**Stage 4 — Charting the Data**

The data will be extracted into a Microsoft Office Excel Spreadsheet and will be categorized using the following variables/categories:

- Title
- Author
- Publication Year
- Population/Study Unit
- Geographical Location/Region
- Study Design etc.

Furthermore, to clearly understand the data extracted from articles, results will be categorised using the five levels of the socio-ecological framework with respect to the Nigerian context. This theoretical framework will be adopted for this study because it describes the complex relationship between individuals and social systems and also aims to suggest the consequence of the interrelationship between an individual’s health behaviour or outcome and their immediate environment (16). The extraction form will consist of supplementary fields to allow for flexibility should any other variable that the researchers did not initially consider come up. The data form will continuously be updated as data extraction is expected to be an iterative process.

**Stage 5 — Collating and Summarizing Results**

With the use of tables and diagrams, results of the review will be presented and will be accompanied by a summary described in a descriptive or narrative format that aligns with the objectives and scope of the review. This will allow for easy accessibility and understanding of findings or results. This review will adapt the PRISMA-ScR checklist as a guideline to ensure that all relevant study and information are captured. There will be no quality assessment as this review is not a systematic review. Also, no ethical approval will be sort for this research as no individual datasets are involved rather this scoping review will only synthesize evidence from articles/literature.

# Acknowledgement

The authors would like to acknowledge the MSc in Public Health Department in the Faculty of Education and Health Sciences in the University of Limerick.

# References

1. OECD. Society at a Glance: Asia/Pacific 20222022.

2. UN-IGME. Child Mortality Estimates. 2021.

3. Hug L, Alexander M, You D, Alkema L. National, regional, and global levels and trends in neonatal mortality between 1990 and 2017, with scenario-based projections to 2030: a systematic analysis. The Lancet global health. 2019;7(6):e710-e20.

4. UNICEF. Under-five mortality 2019 [cited 2020. United Nations Inter-agency Group for Child Mortality Estimation (UN IGME) 2020.]. Available from: <https://data.unicef.org/topic/child-survival/under-five-mortality/>.

5. UN. Transforming our World: The 2030 Agenda for Sustainable Development. Publication. 2015 2015. Contract No.: A/RES/70/1.

6. Salawu MM, Afolabi RF, Gbadebo BM, Salawu AT, Fagbamigbe AF, Adebowale AS. Preventable multiple high-risk birth behaviour and infant survival in Nigeria. BMC Pregnancy and Childbirth. 2021;21(1).

7. USAID. Nigeria Global Health 2021 [Available from: <https://www.usaid.gov/nigeria/global-health>.

8. Ayoade MA. Trends and temporal patterns of infant mortality in Nigeria. GeoJournal. 2020;86(4):1835-48.

9. Matteson DW, Burr JA, Marshall JR. Infant mortality: A multi-level analysis of individual and community risk factors. Social science & medicine (1982). 1998;47(11):1841-54.

10. Upadhyay RP, Krishnan A, Rai SK, Chinnakali P, Odukoya O. Need to Focus Beyond the Medical Causes: a Systematic Review of the Social Factors Affecting Neonatal Deaths. Paediatric and Perinatal Epidemiology. 2014;28(2):127-37.

11. Louangpradith V, Yamamoto E, Inthaphatha S, Phoummalaysith B, Kariya T, Saw YM, et al. Trends and risk factors for infant mortality in the Lao People’s Democratic Republic. Scientific Reports. 2020;10(1):21723.

12. Bugelli A, Borgès Da Silva R, Dowbor L, Sicotte C. Health capabilities and the determinants of infant mortality in Brazil, 2004–2015: an innovative methodological framework. BMC Public Health. 2021;21(1):831.

13. Wallace LA, Rucks AC, Ginter PM, Katholi CR. Social factors and public policies associated with state infant mortality rates. Women & Health. 2021;61(4):337-44.

14. Arksey H, O'Malley L. Scoping studies: towards a methodological framework. International Journal of Social Research Methodology. 2005;8(1):19-32.

15. Tricco AC, Lillie E, Zarin W, O'Brien K, Colquhoun H, Kastner M, et al. A scoping review on the conduct and reporting of scoping reviews. BMC Med Res Methodol. 2016;16:15.

16. Bronfenbrenner U. Toward an experimental ecology of human development. The American psychologist. 1977;32(7):513-31.
