# Supplementary material for: Factors associated with infant mortality in Nigeria: A scoping review
Source: PLoS One. 2023 Nov 15;18(11):e0294434. doi: 10.1371/journal.pone.0294434 (PMC10650982; doi:10.1371/journal.pone.0294434)
Supplement: Attachment — Submitted filename: Rebuttal Letter_PLOS One.docx [file pone.0294434.s005.docx]

Loveth Nwanze

University of Limerick

08 September 2023

The Academic Editor

PLOS ONE

Dear Kahsu Gebrekidan,

We would like to express our sincere appreciation to you and the esteemed reviewers for dedicating your valuable time to review our manuscript. Your constructive feedback has been instrumental in improving the quality of our work. We have carefully considered each of the points raised and have charted the responses and revisions in the table below, with the reviewers' feedback in black and our authors' reply in blue.

We believe that these revisions address the concerns raised by the reviewers and significantly enhance the overall quality and clarity of our manuscript. We sincerely appreciate your guidance and feedback, which have been instrumental in refining our work. We look forward to your further guidance during the subsequent review process and hope that our revised manuscript will meet the high standards of your esteemed journal.

Thank you once again for your valuable input and consideration.

Sincerely,

Loveth Nwanze

University of Limerick

lovnwanze@gmail.com

| **Reviewer** | **Section** | **Reviewer's Comments & Corrections** | **Page No** |
| --- | --- | --- | --- |
| Reviewer #2 | Title | **The title require minor but very important modifications representing correct syntax for such a study and appropriate alignment with the study design. The suggested modification is "Factors associated with infant mortality in Nigeria: A scoping review."** |  |
|  |  | The title now reads “Factors associated with infant mortality in Nigeria: A scoping review” | Title page |
| Reviewer #2 | Abstract | **The abstract should be structured as Background, Objective, Methods and Findings.** |  |
|  |  | The abstract was edited based on the recommendation by the reviewer.  Abstract  **Background** : Infant mortality persists as a global public health concern, particularly in lower-middle-income countries (LIMCs) such as Nigeria. The risk of an infant dying before attaining one year of age is estimated to be six times higher in Africa than in Europe. Nigeria recorded an infant mortality rate of 72.2 deaths per 1,000 live births in 2020, in contrast to the global estimate of 27.4 per 1,000 live births. Several studies have been undertaken to determine the factors influencing infant mortality.  **Objective:** This scoping review sought to identify and summarise the breadth of evidence available on factors associated with infant mortality in Nigeria.  **Methods:** This review followed the five-stage principles of Arksey and O’Malley’s framework. Four electronic databases were searched with no limit to publication date or study type: Ovid MEDLINE, PubMed, CINAHL Complete, and Web of Science. Selected studies were imported into Endnote software and then exported to Rayyan software where duplicates were removed. Included articles were thematically analysed and synthesised using the socioecological model.  **Results:** A total of 8,139 references were compiled and screened. Forty-eight articles were included in the final review. At the individual level, maternal- and child-related factors were revealed to influence infant mortality; socioeconomic and sociocultural factors at the interpersonal level; provision and utilisation of health services, health workforce, hospital resources and access to health services at the organisational level; housing/neighbourhood and environmental factors at the community level; and lastly, governmental factors were found to affect infant mortality at the public policy level.  **Conclusion** : Factors related to the individual, interpersonal, organisational, community and public policy levels were associated with infant mortality in Nigeria.  **Keywords:** newborn, neonatal, infant mortality, Nigeria, scoping review | Pg 1 |
| Reviewer #2 | Abstract | **Editorial corrections are recommended in the abstract. For example, to maintain consistency in methodological syntax, the second and third sentences should read thus; "The risk of an infant dying before attaining one year of age is estimated to be six times higher in Africa than in Europe. Nigeria recorded an infant mortality rate of 72.2 deaths per 1,000 live births in 2020 in contrast with global estimate of 27.4 per 1,000 live births".** |  |
|  |  | **The abstract was edited, and the sentence now reads “**The risk of an infant dying before one year of age is estimated to be six times higher in Africa than in Europe. Nigeria recorded an infant mortality rate of 72.2 deaths per 1,000 live births in 2020, in contrast to the global estimate of 27.4 per 1,000 live births.” | Pg 1 |
| Reviewer #2 | Abstract | **The next statement related to the objective of the study would benefit from a modification to read thus: "This scoping review sought to identify and summarise the breadth of evidence available on factors associated with infant mortality in Nigeria".** |  |
|  |  | The statement about the study's objective was corrected to ensure clarity and accuracy: "This scoping review sought to identify and summarise the breadth of evidence available on factors associated with infant mortality in Nigeria. " | Pg 1 |
| Reviewer #2 | Abstract | **The last sentence of the abstract should read: "Factors related to the individual, interpersonal, organisational, community and public policy levels were associated with infant mortality in Nigeria."** |  |
|  |  | Thank you for your comments. The sentence was edited to ensure clarity: "Factors related to the individual, interpersonal, organisational, community and public policy levels were associated with infant mortality in Nigeria. | Pg 1 |
| Reviewer #2 | Introduction | **The second sentence should read: "The number of infants that die before attaining the age of one year for every 1,000 live births in a year is known as Infant Mortality Rate (IMR)** |  |
|  |  | The sentence now reads:"The number of infants that die before attaining the age of one year for every 1,000 live births in a year is known as the Infant Mortality Rate (IMR)" . | Pg 2 |
| Editor | Introduction | **“Several studies have traced the high mortality rates of infants in Nigeria to demographic and socio-economic factors, the most common being poverty/socioeconomic status and poor health service delivery (9, 11)” This sentence refers to several studies, yet only two studies are referenced. Kindly rephrase the sentence if only two studies are available** |  |
|  |  | The sentence was rephrased thus: " Studies have traced the high mortality rates of infants to demographic and socio-economic factors, the most common being poverty/socioeconomic status and poor health service delivery (11, 12) | Pg 3 |
| Editor | Introduction | **“They revealed that a delay in seeking care (level 1 delay) and a delay in receiving quality treatment at a health facility (level 3 delay) were significant contributors to neonatal mortality compared to delay in reaching a health facility (level 2 delay).” Avoid the use of personalizing pronouns. Please rephrase.** |  |
|  |  | The sentence was edited to follow the feedback from reviewers: "To estimate the burden of societal factors on neonatal deaths, Upadhyay et al. adopted the three-delays model in their study. " | Pg 3 |
| Reviewer #2 | Methodology | **The use of abbreviation PCC in section 2.1 Defining the research question should have been written fully. This should be "The Population-Concept-Context (PCC) framework applied defines the scope as infants mortality in the context of Nigeria".** |  |
|  |  | “The Population, Concept, and Context (PCC) framework guided the inclusion criteria for this review”. | Pg 4 |
| Reviewer #1 | Methodology | **Identifying relevant literature: The authors searched in social sciences databases Sabinet Africa and Africabib besides four other electronic databases—PubMed Ovid MEDLINE, CINAHL Complete, and Web of Science . However, the authors need a search in other online databases as a number of Nigerian studies may not be found on these sites. Examples are the African index Medicus, African Journal online (AJOL) and even Google scholar. Utilizing these sources will show the authors did an extensive and exhaustive search.** |  |
|  |  | We appreciate the recommendation to broaden our search sources. However, the authors had previously searched African Journal online (AJOL) during the initial study identification but did not utilise the database as there was no provision for advanced search to utilise the proposed search strategy. Also, we did not see the need to include the other mentioned databases as the ones used for the study provided a comprehensive literature review and scope. | Pg 5 |
| Reviewer #1 | Methodology | **Eight studies were excluded as they were not written in English, did the authors attempt to translate it with an translator app to see whether those studies were relevant? It is suggested that the authors use an app to translate to check for relevance and fi onto inclusion criteria.** |  |
|  |  | The inclusion criteria set by the authors was to exclude articles written in any language other than English, as this is the official language used in Nigeria. | ? |
| Reviewer #2 | Methodology | **The Data Extraction Chart showing studies are arranged in no order. Kindly redraw based on year (latest to earliest or vice versa)** |  |
|  |  | The data extraction chart has been reordered based on publication year, with the oldest studies appearing first. | Pg 9-12 |
| Reviewer #1 | Results | **All figures are not labeled appropriately. Titles are within the result while figures without Titles are after references. Kindly address** |  |
|  |  | According to the submission guidelines, figures are not to be added to the main manuscript, but rather, a figure caption must be inserted in the manuscript's text. The titles seen after the reference are for supplementary information and not for the figures, which are in separate attachments. The titles for tables and figures were checked, and they follow the journal submission guidelines. | Pg 8 and 18 |
| Reviewer #1 | Results | **Page 16 to 18 prose is a complete repeat of the contents of Table 3. Please avoid repetition. Present the prose in results with appropriate reference to the Table.** |  |
|  |  | Thank you for the feedback. Pages 16 and 17 were edited to ensure no repetition and the results were presented appropriately. | Pg 16-17 |
| Reviewer #1 | Discussion | **The authors did not compare the factors identified in some instances with other studies nor proffer reasons for these findings (birth outcomes, immunization).** |  |
|  |  | The discussion sections were re-edited to ensure adequate discussion of the results and the proper contextualisation in the existing literature. | Pg 17-33 |
| Reviewer #1 | Discussion | **Also, the author should try and relate these factors to the Nigerian statistics. For instance, what is the proportion of polygynous marriages, educated females, immunisation in Nigeria? And how those this relate to the finding with the infant mortality. Do the same thing for the other factors. These may be contributing reasons towards the slow decline in infant mortality.** |  |
|  |  | We have incorporated relevant Nigerian statistics into the discussion of identified factors, to improve the contextualisation of the findings in the Nigerian context. | Pg 17-33 |
| Reviewer #1 | Discussion | **The paragraphs are too long and the authors often discuss more than one factor in a paragraph making it hard to read. Kindly re-organize.** |  |
|  |  | The discussion section was edited to ensure clarity and the appropriate length of sentences and paragraphs to improve the academic writing, the flow and the clarity of the discussion section. | Pg 17-33 |
| Reviewer #1 | References | **The authors need to address errors in titles like all capital letters (4,22, 63) or first letters of words as capitals (17, 22,23, 27, 32,46, 50,52,53, 62,64,71. 74,81, 85, 86, 88, 92, 93, 96 and, repeats of author name (25) or missing journal name (64)** |  |
|  |  | The errors in the references section were checked and corrected in the new document. 4. Frey R, Cui W. Infant mortality in the world-system. Journal of Globalization Studies. 2016;7:47-55. 23. Barfield WD, Committee On F, Newborn. Standard terminology for fetal, Infant, and perinatal deaths. Pediatrics. 2016;137(5). 73. Cyril O, Mathias B. Private health care finance and infant mortality rate in Nigeria. Nigerian Studies in Economics and Management Sciences. 2022;4:10-8. * repeats of author name (25) now 31. Uthman OA, Uthman MB, Yahaya I. A population-based study of effect of multiple birth on infant mortality in Nigeria. BMC Pregnancy Childbirth. 2008;8:41. * Missing journal name (64) now 73. Cyril O, Mathias B. Private health care finance and infant mortality rate in Nigeria. Nigerian Studies in Economics and Management Sciences. 2022;4:10-8. * All errors related to the following references (17, 22,23, 27, 32,46, 50,52,53, 62,64,71. 74,81, 85, 86, 88, 92, 93, 96) have been corrected. New numbers are (18, 23, 24, 53, 47, 50, 51, 37, 63, 52, 73, 70, 75, 108, 111, 95, 116, 96, 120, 121). | Pg 35-40 |
