# Supplementary material for: Factors associated with infant mortality in Nigeria: A scoping review
Source: PLoS One. 2023 Nov 15;18(11):e0294434. doi: 10.1371/journal.pone.0294434 (PMC10650982; doi:10.1371/journal.pone.0294434)
Supplement: Attachment — Submitted filename: PONE D 2305730R1 Rebuttal Letter 2.docx [file pone.0294434.s007.docx]

Loveth Nwanze

University of Limerick

Ireland.

12 October 2023

The Academic Editor,

PLOS ONE

Dear Kahsu Gebrekidan,

We would like to extend our heartfelt appreciation to both you and the esteemed reviewers for generously dedicating your valuable time to reviewing our manuscript and providing invaluable feedback. Your meticulous evaluation has played a pivotal role in enhancing our submitted paper's quality and clarity, particularly, the final comments helped improve the methodology and limitation sections.

We have taken great care to thoroughly address each of the points raised, and we have documented our responses and revisions in the table below, with the reviewers' feedback indicated in black and our corresponding author responses highlighted in blue. These revisions have been strategically made to align with the reviewers' concerns, ultimately significantly improving the overall quality and clarity of our methodology and limitations sections.

We eagerly anticipate your continued guidance throughout the subsequent stages of the review process, and we are committed to ensuring that our revised manuscript upholds the rigorous standards of your esteemed journal.

Once again, we extend our gratitude for your invaluable input and consideration.

Kind Regards,

Loveth Nwanze

| **COMMENTS** | **SECTION** | **REVIEWER'S COMMENTS & CORRECTIONS** | **PAGE NO** |
| --- | --- | --- | --- |
| **Comment 1** | **Methodology and Limitation** | **The authors have made most of the changes/corrections suggested and addressed most of the reviewer’s concerns. However, the inclusion criteria of the authors does not state the use of articles published only in English (check the PCC segment of methodology)** |  |
|  |  | Thank you for your feedback and for highlighting the critical missing inclusion criteria. We have revised the sections to enhance the clarity of the inclusion and exclusion criteria, including our decision to consider articles published in English exclusively. This choice aims to facilitate the research team's ability to thoroughly review and synthesise the articles to address the research question effectively.  However, it is essential to acknowledge that this decision to include only English-language articles introduces a potential limitation by inadvertently introducing language bias.  Please find below the edited section on the page.  “Furthermore, studies conducted only in settings outside the Nigerian context and those involving animal subjects were not included in this review. The study exclusively encompassed articles that have been published in English.” | Page 6 |
| **Comment 2** | **Methodology and Limitation** | **The reviewer suggested checking for other articles not written in English as long as they have all the MESH terms used in the search strategy. The authors excluded it based on English being the official language of the country. Authors should note that publications about Nigeria could be made in non-English speaking countries/journals! Furthermore, the authors included Sub-Saharan African countries that mentioned Nigeria in the context of their search. Not all Sub-Saharan countries are English speaking, and this may result in missing out studies that had Nigeria inclusive.** |  |
|  |  | Thank you very much for your comment. We sincerely apologise for the oversight in our previous responses despite our careful efforts to address all corrections suggested by the editor and reviewers. The decision to exclude articles related to Nigerian Infant Mortality published in languages other than English, such as Spanish, French, Chinese, etc., was driven by the research team's limited ability to read and understand articles in other languages. While this constraint was unavoidable, we acknowledge that it should have been explicitly mentioned in the limitations section of the manuscript. Consequently, we have rectified this omission by adding a section in the limitations segment of the paper to acknowledge that including articles solely published in English represents one of the research limitations.  Please find the updated limitation section below:  “Despite these strengths, this review did not report on medical causes and genetic risk factors because the model only allows for categorisations based on social and environmental inter-relationships. Another limitation of the study is its exclusive inclusion of articles published solely in English. Lastly, this review did not make use of a quality assessment tool, and as such, did not critically appraise the articles included, however, this also supports the review’s objective, which is to provide an overview of existing and current studies on infant mortality in Nigeria.”  Thank you once again for bringing this to our attention, and we appreciate your understanding in this matter. | Page 35 |
| Comment 3 | Methodology and Limitation | **3. There is a conflict between these two statements underlined in the inclusion and exclusion criteria, respectively, kindly reconcile.’ The context for the review was Nigeria. In very few cases, studies within the context of Sub-Saharan Africa (SSA) were included if they provided precise results regarding IMR in Nigeria. Studies on animal subjects were excluded, including studies with participants and study settings from a country other than Nigeria.** |  |
|  |  | We appreciate your comment as it contributes to the clarity of our inclusion and exclusion criteria. The criteria have been revised to provide a more precise context for the included articles and to eliminate any potential source of confusion. Our aim has been to ensure clarity and consistency throughout all sections. The sentence now reads thus:  “The geographic scope of this review was limited to Nigeria. In instances where a study encompassed multiple countries, including Nigeria, we included articles only if it was possible to extract and differentiate information specifically pertaining to Nigeria from the data related to the other countries.  In addition to the above-stated inclusion criteria, no restriction was imposed on publication dates. This flexibility was integral to our objective of comprehensively mapping and summarising the determinants of infant mortality in Nigeria”. | Page 6 |
