# Supplementary material for: Factors associated with infant mortality in Nigeria: A scoping review
Source: PLoS One. 2023 Nov 15;18(11):e0294434. doi: 10.1371/journal.pone.0294434 (PMC10650982; doi:10.1371/journal.pone.0294434)
Supplement: Attachment — Submitted filename: Reviewer Comments PLOS.docx [file pone.0294434.s004.docx]

General- The subject matter is important and the relevance has been demonstrated by the authors in the introduction segment. The authors however need to do some more searches of some databases specific for African literature. The authors have mixed UK and American English spelling.

Abstract- Reads well

Introduction-

- Be consistent in use of decimals, either two or one, preferably one in the text.
- “Despite being one of the largest economies in Africa, with an estimated GDP of $448 billion in 2019, and a population of over 200 million people, Nigeria keeps dragging back Africa’s socioeconomic indicators, especially child mortality.” What is the role of the population in this statement? It will read better if removed. Kindly rephrase, the underlined aspect ( e,g the socio-economic indicators of Nigeria remains low).
- “Several studies have traced the high mortality rates of infants in Nigeria to demographic and

socio-economic factors, the most common being poverty/socioeconomic status and poor

health service delivery (9, 11)” This sentence refers to several studies, yet only two studies are referenced. Kindly rephrase the sentence if only two studies are available.

- “Other studies have associated the factors affecting infant mortality with mother and child characteristics.” The authors refer to studies in Nigeria in the previous statement regarding cause of high mortality but the studies referenced concerning mother and child characteristics are not Nigerian studies (Ref 12-15). The authors are trying to highlight factors associated with high IMR so they should do these without attributing it to Nigeria in the introduction as this is the reason for their study.
- “They revealed that a delay in seeking care (level 1 delay) and a delay in receiving quality treatment at a health facility (level 3 delay) were significant contributors to neonatal mortality compared to delay in reaching a health facility (level 2 delay).” Avoid the use of personalizing pronouns. Please rephrase.

Methodology

- Identifying relevant literature: The authors searched in social sciences databases Sabinet Africa and Africabib besides four other electronic databases—PubMed Ovid MEDLINE, CINAHL Complete, and Web of Science . However, the authors need a search in other online databases as a number of Nigerian studies may not be found on these sites. Examples are the African index Medicus, African Journal online (AJOL) and even Google scholar. Utilizing these sources will show the authors did an extensive and exhaustive search.
- Eight studies were excluded as they were not written in English, did the authors attempt to translate it with an translator app to see whether those studies were relevant? It is suggested that the authors use an app to translate to check for relevance and fi onto inclusion criteria.
- The Data Extraction Chart showing studies are arranged in no order. Kindly redraw based on year (latest to earliest or vice versa)

Results

- All figures are not labeled appropriately. Titles are within the result while figures without Titles are after references. Kindly address.
- Page 16 to 18 prose is a complete repeat of the contents of Table 3. Please avoid repetition. Present the prose in results with appropriate reference to the Table.

Discussion

- The authors did not compare the factors identified in some instances with other studies nor proffer reasons for these findings (birth outcomes, immunization).
- Also the author should try and relate these factors to the Nigerian statistics. For instance, what is the proportion of polygynous marriages, educated females, immunization in Nigeria? And how those this relate to the finding with the infant mortality. Do the same thing for the other factors. These may be contributing reasons towards the slow decline in infant mortality.
- The paragraphs are too long and the authors often discuss more than one factor in a paragraph making it hard to read. Kindly re-organize.

References

- The authors need to address errors in titles like all capital letters (4,22, 63) or first letters of words as capitals (17, 22,23, 27, 32,46, 50,52,53, 62,64,71. 74,81, 85, 86, 88, 92, 93, 96 and, repeats of author name (25) or missing journal name (64)
