# Supplementary material for: Factors associated with infant mortality in Nigeria: A scoping review
Source: PLoS One. 2023 Nov 15;18(11):e0294434. doi: 10.1371/journal.pone.0294434 (PMC10650982; doi:10.1371/journal.pone.0294434)
Supplement: Attachment — Submitted filename: PONE D 2305730R1 REVIEWER COMMENTS.docx [file pone.0294434.s006.docx]

- The authors have made most of the changes/corrections suggested and addressed most of the reviewer’s concerns. However, the inclusion criteria of the authors does not state the use of articles published only in English (check the PCC segment of methodology)
- The reviewer suggested checking for other articles that are not written in English as long as it has all the MESH terms used in the search strategy. The authors excluded it based on English being the official language of the country. Authors should note that publications about Nigeria could be made in non-English speaking countries/journals! Furthermore, the authors included Sub-Saharan African countries that mentioned Nigeria in the context of their search. Not all Sub-Saharan countries are English speaking, and this may result in missing out studies that had Nigeria inclusive.
- There is a conflict between these two statements underlined in the inclusion and exclusion criteria respectively, kindly reconcile’

The context for the review was Nigeria. In very few cases, studies within the context of

Sub-Saharan Africa (SSA) were included if they provided precise results regarding IMR in Nigeria.

Studies on animal subjects were excluded,

including studies with participants and study settings from a country other than Nigeria.
